# New Computational Approaches to Aging Research

**DOI:** 10.1093/geroni/igaa057.2618

**Published:** 2020-12-16

**Authors:** Morgan E Levine

**Affiliations:** Yale University School of Medicine, New Haven, Connecticut, United States

## Abstract

Aging is associated with numerous changes at all levels of biological organization. Harnessing this information to develop measures that accurately and reliably quantify the biological aging process will require systems biology approaches. This talk will illustrate how epigenetic data can be integrated with cellular, physiological, proteomic, and clinical data to model age-related changes that propagate up the levels—finally manifesting as age-related disease or death. I will also describe how network modeling and machine learning approaches (linear and non-linear) can be used to identify causal features in aging from which to generate novel biomarkers. Given the complexity of the biological aging process, modeling of systems dynamics over time will both lead to the development of better biomarkers of aging, and also inform our conceptualization of how alterations at the molecular level propagate up levels of organization to eventually influence morbidity and mortality risk.

